# Ectopic Expression Reveals a Conserved *PHYB* Homolog in Soybean

**DOI:** 10.1371/journal.pone.0027737

**Published:** 2011-11-16

**Authors:** Fa-Qiang Wu, Xiao-Mei Zhang, Dong-Mei Li, Yong-Fu Fu

**Affiliations:** Institute of Crop Sciences, National Key Facility of Crop Gene Resource and Genetic Improvement, Chinese Academy of Agricultural Sciences, Haidian District, Beijing, China; University of Umeå, Sweden

## Abstract

Phytochromes sense red/far-red light and trigger a cascade of physiological responses in plant. Here, a *phytochrome B* homolog, *GmPHYB1*, was amplified from the soybean genome, and its expression profiles were obtained for various parts of the plant and at various developmental stages. The gene was ectopically expressed in *Arabidopsis thaliana*, driven by CaMV 35S promoter, to study the physiological functions of the gene product. The overexpressors of *GmPHYB1* behaved similarly to those of *AtPHYB*, but with some subtle differences with respect to the acceleration of flowering under short day conditions and the growth of the hypocotyl under certain light fluence rate. The results suggested that this soybean *PHYB* homolog was well conserved both at the level of sequence and physiological function.

## Introduction

For better growth and development, plants have evolved a series of photoreceptors to respond to light. One of the important kinds of photoreceptors is phytochromes (PHY), which are essential for red and far-red light sensing and involved in such responses as seed germination, photomorphogenesis, shade avoidance, flowering, and a number of other adaptive responses [1], [2], [3]. PHY molecules are composed of a linear tetrapyrrole chromophore covalently linked to an apoprotein and function as dimers that undergo a reversible comformational change between the inactive (Pr) and the active (Pfr) form in response to red or far-red light, allowing the phytochrome to act as a switch that is turned on or turned off [4], [5], [6].

The PHY molecules are encoded by a small gene family, and have different members in different plants. The *Arabidopsis thaliana* genome includes five *PHY* genes (*PHYA* to *PHYE*), while the rice genome includes just three (*PHYA* to *PHYC*). Based on phylogenetic analysis, all phytochromes found in plants can be classified into two groups, the *PHYA* branch, including *PHYA* and *PHYC*, and the *PHYB* branch, including *PHYB*, *PHYD* and *PHYE* [7]. The various phytochromes play overlapping, yet distinct roles. In *A. thaliana*, *PHYB* is the principal and primary mediator of red light responses and shade avoidance [8]. It predominates in light-grown plants [9], promoting seed germination and de-etiolation in response to red light, inhibiting shade avoidance responses under a high ratio of red: far-red light (R:FR) [10]. Such responses are important for the plants to survive in the ever-changing environmental conditions during its life cycle.

Some information is also available regarding the *PHY* of the rice [11] and tobacco [12]. However, despite of some report of pea *PHYA* and *PHYB* [13] and soybean *PHYA* [14], [15], there has been limited molecular analysis of *PHYB* in legumes, especially in soybean (*Glycine max* (L.) Merr.), a paleopolyploid species with a complex genome [16].

Here we report the isolation of *GmPHYB1*, a soybean *PHYB* homolog, and the documentation of its expression profiles. The ectopic overexpression of *GmPHYB1* in *A. thaliana* resulted in strong suppression of shade avoidance, early flowering under short days (SD), the shortening of the hypocotyls and the lengthening of the roots. We detail the impact on hypocotyl length of the expression of *GmPHYB1* in *A. thaliana* plants grown under a variety of light fluence rate, and demonstrate that a number of genes related to flowering time and hypocotyl elongation show altered expression profiles. Our results suggested that both the sequence and function of *GmPHYB1* are well conserved across evolutionarily diverse species.

## Results

### The soybean genome includes a *PHYB* homolog

The EST sequence TC227575 represented in the DFCI Soybean Gene Index database (http://compbio.dfci.harvard.edu/tgi/cgi-bin/tgi/gimain.pl?gudb=soybean) was identified as a soybean *PHYB* homolog on the basis of its sequence similarity with *A. thaliana PHYB* (*AtPHYB*). Its sequence was used to design a pair of primers (Table S1) to amplify the soybean *PHYB* gene from a leaf mRNA template extracted from the soybean cultivar Kennong 18. The resulting sequence (hereafter *GmPHYB1*) encoded a predicted 1,137 residue protein which matched the translated Glyma09g03990 product (Phytozome; http://www.phytozome.net/soybean). GmPHYB1 shared a 76% level of peptide identity with AtPHYB, and included all the known functional domains of PHY (Figure S1). The *GmPHYB1* sequence has been submitted to the GenBank/EMBL/DDBJ database (accession number EU428749).

**Figure 1:**
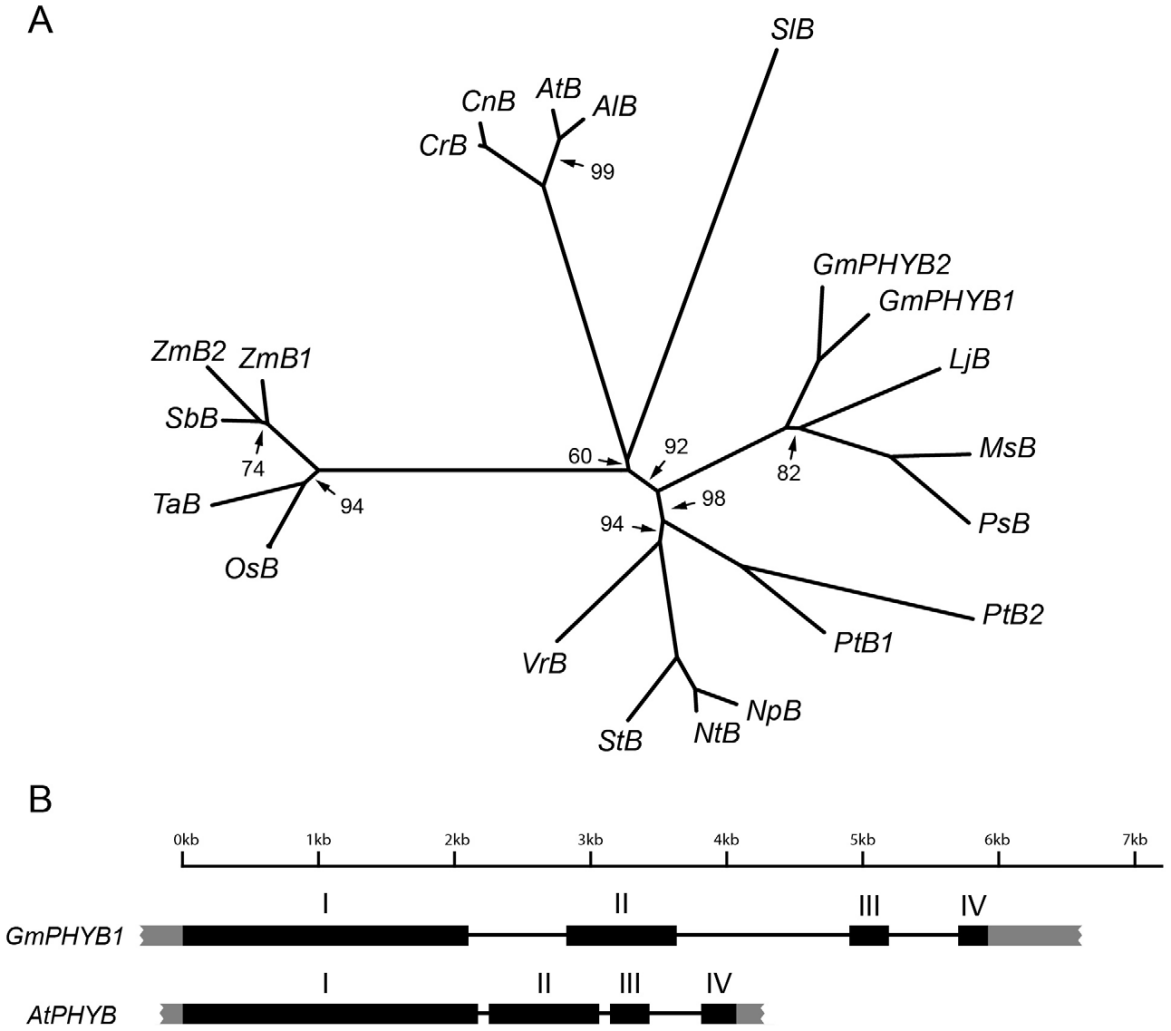
A *PHYB* homolog gene exists in the soybean genome. (A) A neighbor-joining tree constructed with MEGA 4.0 software based on the full-length amino acid sequences. The bootstrap analysis (1000 replicates) was performed. Except for the arrows indicated, all bootstrap values  =  100. Sequences examined are (accession number in parenthesis): *Arabidopsis lyrata PHYB* (*AlB*, Q5G889), *Arabidopsis thaliana PHYB* (*AtB*, P14713), *Cardamine nipponica PHYB* (*CnB*, C4TGD7), *Cardamine resedifolia PHYB*(*CrB*, C4TGE0), *Glycine max PHYB1* (*GmPHYB1*, EU428749) and *PHYB2* (*GmPHYB2,* Glyma15g14980), *Lotus japonicus PHYB* (*LjB*, A1IIA2), *Medicago sativa PHYB* (*MsB*, C7FHN7), *Nicotiana plumbaginifolia PHYB* (*NpB*, O24117), *Nicotiana tabacum PHYB*(*NtB*, P29130), *Oryza sativa* subsp. *Japonica PHYB*, (*OsB*, Q10MG9), *Pisum sativum PHYB*, (*PsB*, Q9SEW2), *Populus trichocarpa PHYB1* (*PtB1*, Q9FPQ3) and *PHYB2* (*PtB2*, Q9FPQ2), *Solanum tuberosum PHYB*, (*StB*, P34094), *Sorghum bicolor PHYB*(*SbB*, P93527), *Stellaria longipes PHYB*, (*SlB*, Q717V7), *Triticum aestivum PHYB* (*TaB*, A9JR06), *Vitis riparia PHYB* (*VrB*, B9U4G7) and *Zea mays PHYB1* (*ZmB1*, Q6XFQ3) and *PHYB2* (*ZmB2*, Q6XFQ2). (B) The gene structure of *GmPHYB1*, compared with that of *AtPHYB* (AT2G18790). Exons are represented by black boxes; introns by lines and UTRs by grey boxes.

A distance-based, neighbor-joining tree constructed by comparing the GmPHYB1 sequence with related peptide sequences (including GmPHYB2, the translated product of another soybean *PHYB* homolog, Glyma15g14980, found by screening the soybean genomic sequence database Phytozome) revealed its relationship with homologs from other plant species (Figure 1A). The tree showed two major clades, one corresponding to the monocotyledonous species, and the other to the dicotyledonous ones. The GmPHYB1 and GmPHYB2 sequences clustered with the latter, within the legume sub-clade alongside *Lotus japonicus*, *Pisum sativum* (alfalfa) and *Pisum sativum* (pea) PHYB homologs, which are particularly closely related to one another as all three are Galegoid legumes, while soybean is a Phaseoloid [17], [18]. Thus interspecific divergence in the PHYB sequence appears to largely reflect phylogenetic relationships.

To determine the structure of *GmPHYB1*, its cDNA sequence was aligned with its corresponding genomic DNA sequence, as retrieved from the Phytozome database. This revealed that the genomic sequence includes four exons (Figure 1B), as also is the case for the *PHYB* genes present in *A. thaliana*, potato [19], tomato[20], rice [11], [21] and maize [22] *PHYB*s. Although the exon lengths in *GmPHYB1* were comparable with those in *AtPHYB*, the soybean introns were considerably larger (Figure 1B).

### The endogenous expression of *GmPHYB1*

**Figure 2:**
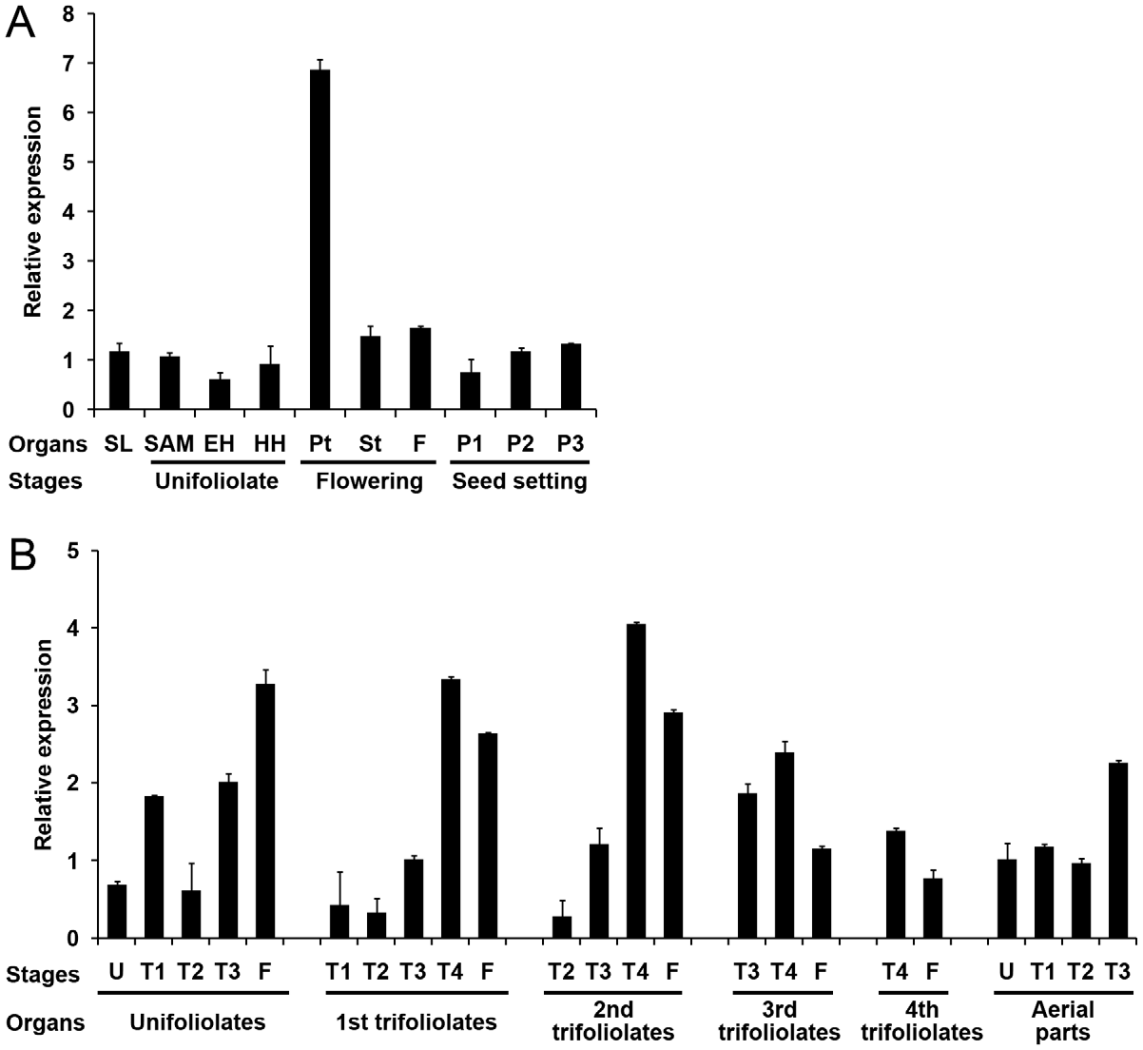
The expression profiles of *GmPHYB1* in various tissues/organs and developmental stages. (A) Tissue/organ expression profiles. SL, seedling; SAM, shoot apex (including the apical meristem and immature leaves); EH, epicotyl; HH, hypocotyl; Pt, petiole; St, stem; F, flower buds; P1 to P3, pods (excluding seeds) at 7, 14, and 21 days after flowering. Stages defined as follows: unifoliolates, unifoliolates fully opened; flowering, onset of flowering; seed setting, initiation of seed growth. (B) Expression profiles in leaves and aerial parts of the plant at different developmental stages. U and T1–T4 indicated different leaves or aerial parts (Organs) when leaves opened fully (Stages). U, fully opened unifoliolates; T1, fully opened 1^st^ trifoliolates; T2, fully opened 2^nd^ trifoliolates; T3, fully opened 3^rd^ trifoliolates; T4, fully opened 4^th^ trifoliolates; F, flowering. Error bars denote the standard deviation.

The transcription of *GmPHYB1* in various tissues/organs of soybean cv. Kennong 18 plants grown under SD conditions at 28°C for 12 days was assessed using quantitative real-time RT-PCR (qPCR) (Figure 2A). *GmPHYB1* transcript was present in all of the sampled organs/tissues, consistent with the pattern of *PHYB* expression in *A. thaliana* and potato [19], [23], where the gene appears to be expressed in most parts of plants. *GmPHYB1* was expressed at a relatively high level in the petiole, suggesting that it might have an important role in petiole elongation, as shown similarly for *A. thaliana* that phytochrome regulated petiole elongation of *Arabidopsis* [24]. Transcript abundance increased gradually from day 7 to day 21 after flowering, the period during which the pod matured.

The patterns of expression in the leaf and aerial parts of the plant at different developmental stages are shown in Figure 2B. The level of *GmPHYB1* transcript was low in the unifoliolates and the 1^st^ and 2^nd^ trifoliolates, but increased gradually thereafter, reaching a peak of expression just prior to flowering. At the later developmental stages, the level was higher in the unifoliolates and the 1^st^ and 2^nd^ trifoliolates than in the 3^rd^ and 4^th^ trifoliolates. The level of expression in the aerial part of the plant was modest and rather constant until the full expansion of the 3^rd^ trifoliolate.

### The ectopic expression of *GmPHYB1* in *A. thaliana* alters plant architecture

**Figure 3:**
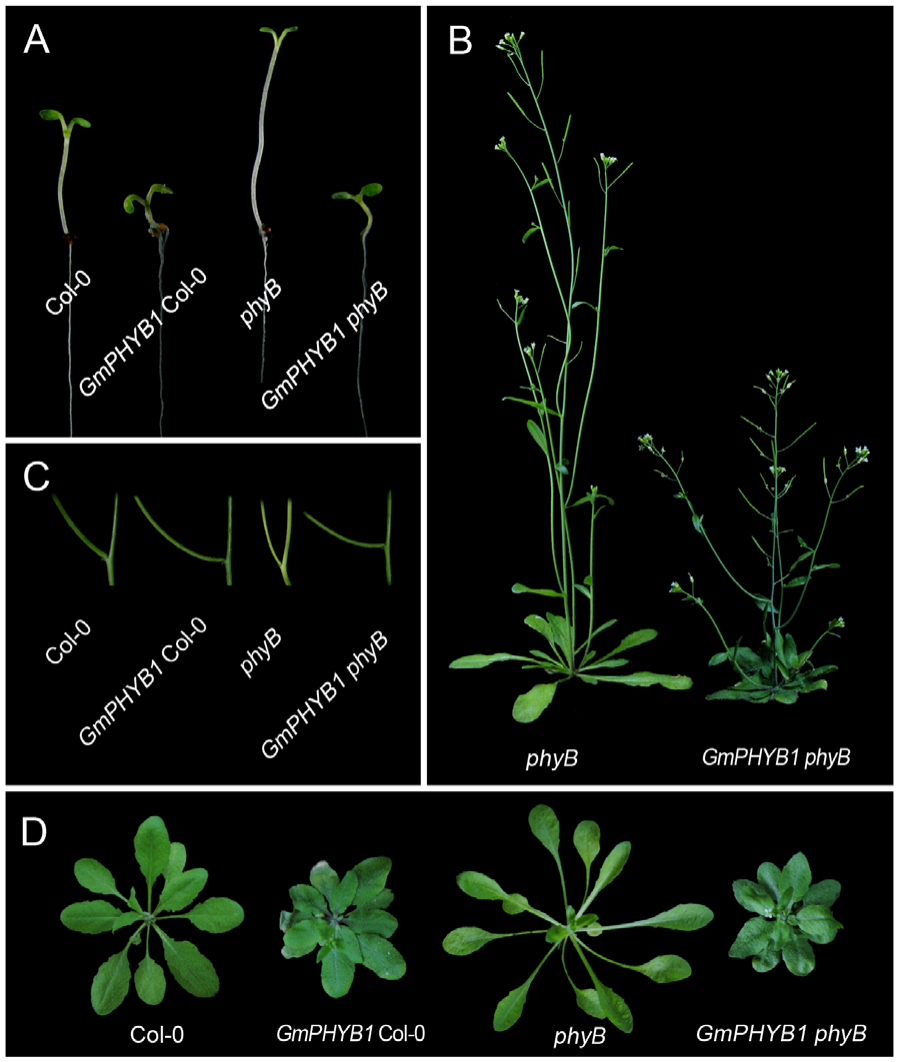
Altered stature of *A. thaliana* plants overexpressing *GmPHYB1*. (A) Strong inhibition of hypocotyl length; (B) compact, semi-dwarf architecture with shortened petioles and floral stems; (C) increased angle between the main inflorescence and the lateral branch; (D) deep green leaf color, larger leaf size and shortened petioles.

PHYB contributes most strongly to shade avoidance responses [8]. The function of GmPHYB1 was explored by its overexpression driven by the CaMV 35S promoter in both wild type *A. thaliana* ecotype Col-0 and in the corresponding loss-of-function mutant *phyB* (*phyB-9*). The seedling stage of both transgenic lines developed a markedly shortened hypocotyl (Figure 3A). The ectopic expression of *GmPHYB1* not only rescued the phenotype of the *phyB* mutant, but also strongly suppressed the plants' shade avoidance phenotype. On the whole, the transgenic plants, regardless of its background, showed a compact, semi-dwarf stature with the shortened petioles and floral stems (Figure 3B). In addition, overexpression led to prostrate rosette leaves, a deep green leaf color and larger leaf size (compared to the *phyB* mutant), and an increased branching angle between the main inflorescences and the petioles or the lateral branches (Figure 3C, D). A similar phenotype has been associated with *PHYB* overexpressors in *A. thaliana* [25], potato [26] and tobacco [27], consistent with a high degree of functional conservation among disparate *PHYB* homologs.

### The ectopic expression of *GmPHYB1* accelerates flowering in *A. thaliana* under SD, but not under LD

Floral transition is regulated by environmental stimuli via signaling cascades [28]. Phytochromes perceive the light signal and play an important role in photoperiodic flowering [29]. The influence of GmPHYB1 on the regulation of flowering time was studied by measuring leaf number and flowering time in transgenic *A. thaliana* overexpressing *GmPHYB1* grown under either long days (LD) or short days (SD).

A set of 18 independent Col-0 and 25 *phyB* transgenics was obtained. Within each of the two groups, all lines behaved consistently with respect to their flowering time (as well as for hypocotyl and root length, as discussed below), so a single line has been used from each group (referred to, respectively, as *GmPHYB1* Col-0 and *GmPHYB1 phyB*) to present the data. Meanwhile, a matching pair of *A. thaliana* transgenic lines in which *AtPHYB* was expressed driven by the CaMV 35S promoter (referred to, respectively, as *AtPHYB* Col-0 and *AtPHYB phyB*) represented a control. The expression level of *GmPHYB1* and *AtPHYB* gene in these plants was confirmed by qPCR (Figure S2).

**Figure 4:**
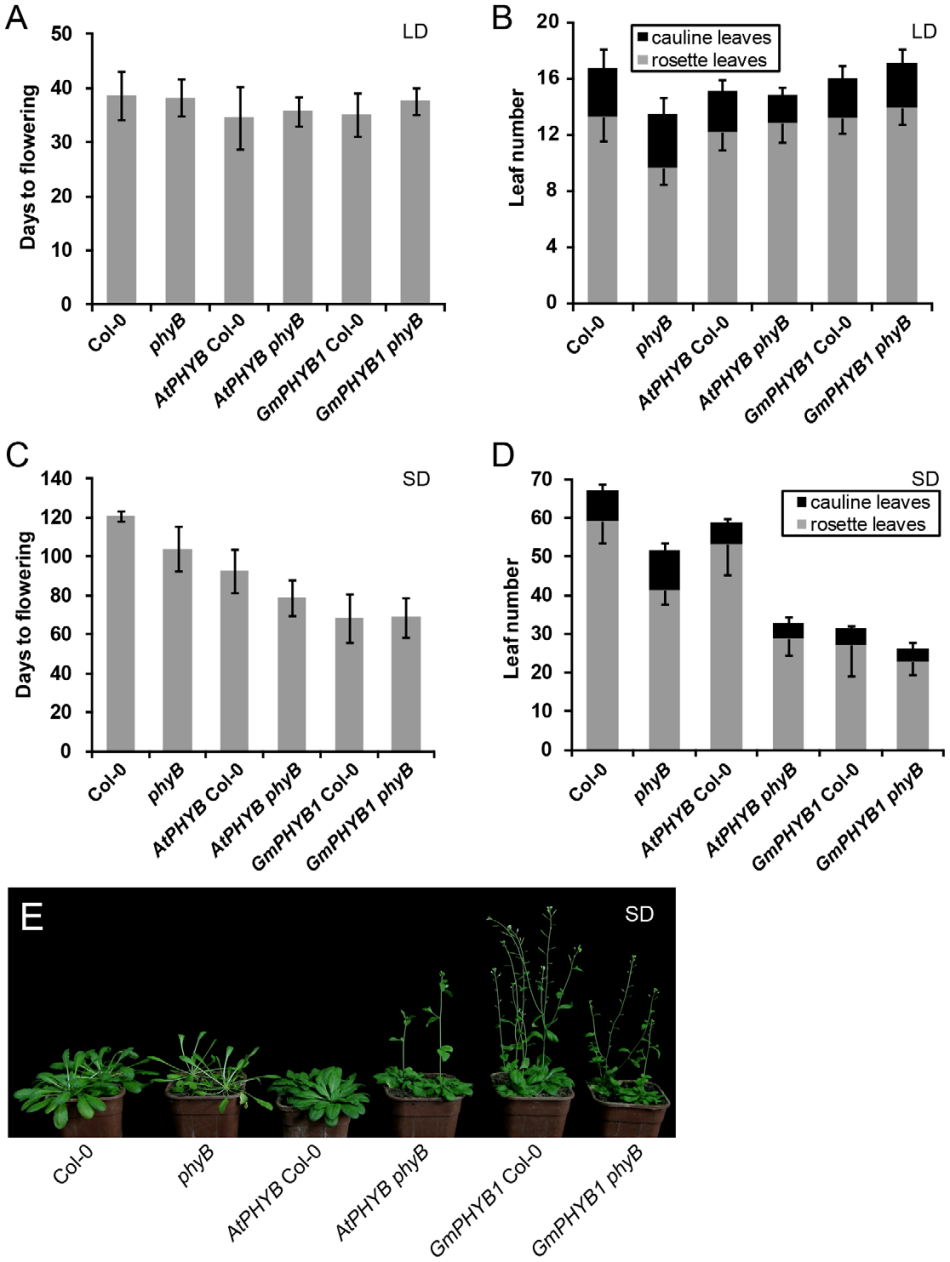
The overexpression *GmPHYB1* accelerates flowering under SD, but not under LD. Leaf number (A, C) and number of days to flowering (B, D) under LD (A, B) and SD (C, D). Error bars denote the standard deviation; n = 20 plants. (E) Flowering phenotypes of the overexpressors of *GmPHYB1* and *AtPHYB* under SD conditions.

Under LD conditions, there was little variation with respect to flowering time among the four transgenic lines and the two non-transgenics Col-0 and *phyB* (Figure 4A). The *phyB* mutant produced fewer rosette leaves and more cauline leaves than Col-0. The rosette leaf number formed by the *AtPHYB* overexpressor and the *GmPHYB1* overexpressor was closer to that formed by Col-0 than by *phyB* (Figure 4B), suggesting that the expression of *GmPHYB1* was able to rescue the mutant phenotype.

Under SD conditions (Figure 4C), both the mutant and the transgenics flowered earlier than Col-0 (Figure 4E), but the flowering time of the transgenics was background-dependent. *AtPHYB phyB* plants flowered earlier than *AtPHYB Col-0* ones, while the *GmPHYB1* overexpressors in both backgrounds flowered earlier than either of the *AtPHYB* overexpressors. The leaf number under SD conditions (Figure 4D) was incompletely correlated with days to flowering (Figure 4C). For example, although *AtPHYB* Col-0 produced more rosette leaves than *phyB*, it nevertheless flowered earlier. It was noteworthy that the number of rosette leaves formed by *AtPHYB* Col-0 was almost double that formed by *AtPHYB phyB*, but there was little difference in rosette leaf number between *GmPHYB1* Col-0 and *GmPHYB1 phyB* (Figure 4D).

### *GmPHYB1* mediates de-etiolation responses in *A. thaliana* under red light

In *A. thaliana, PHY* promotes de-etiolation in response to light, and hypocotyl elongation is considered the standard test of this light-responsiveness [10], [30]. The effect of GmPHYB1 on this trait was tested by exposing seedlings of the set of four transgenic and two non-transgenic lines for five days to darkness, far-red, blue, red or white light.

**Figure 5:**
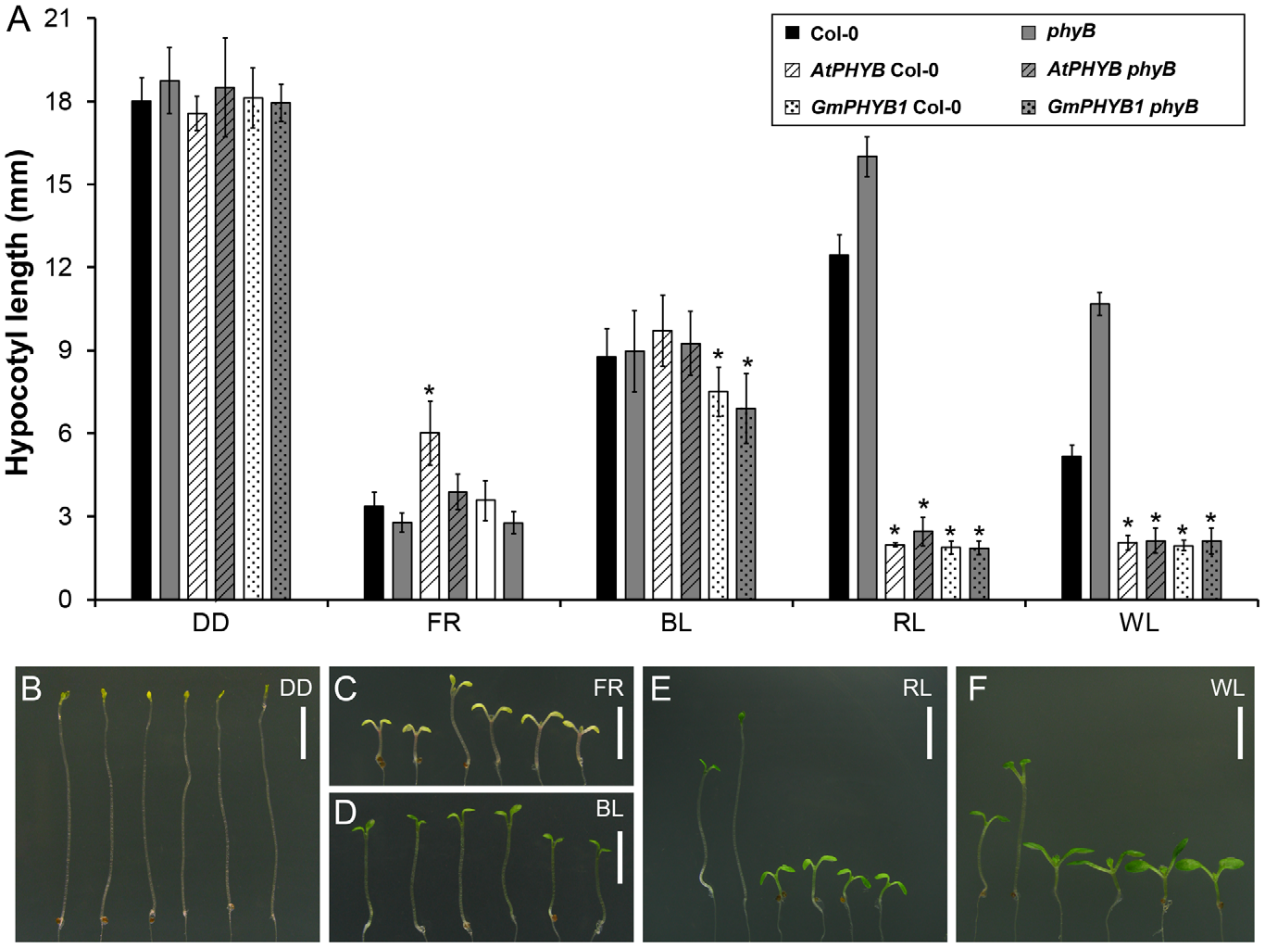
Hypocotyl length of transgenic plants under different light qualities. (A) Five-day-old seedlings of Col-0, *phyB*, *AtPHYB* Col-0, *AtPHYB phyB*, *GmPHYB1* Col-0 and *GmPHYB1 phyB* grown under DD, FR (23.5 µmol m^−2^ s^−1^), RL (1.3 µmol m^−2^ s^−^), BL (3.1 µmol m^−2^ s^−1^) or WL (80.8 µmol m^−2^ s^−1^) conditions. Asterisks indicated a 0.01 significant difference from its transgenic background (t-test). Error bars denote the standard deviation. n = 15. (B–F) Images of the corresponding seedlings (from left to right in each panel: Col-0, *phyB*, *AtPHYB* Col-0, *AtPHYB phyB*, *GmPHYB1* Col-0, *GmPHYB1 phyB*). Bar  =  0.5 cm.

Under the dark (DD) conditions, the six lines behaved indistinguishably, producing a so-called ‘skotomorphogenic’ phenotype [28] characterized by a long hypocotyl and closed, non-expanded yellow cotyledons (Figure 5A and Figure 5B).

Under far-red light (FR), the cotyledons of all the lines remained yellow, but hypocotyl length was clearly inhibited and the cotyledons were open and expanded (Figure 5A, 5C), consistent with the function of endogenous PHYA under far-red light. *AtPHYB* Col-0 seedlings produced a longer hypocotyl than any of the other lines (see later discussion below).

When exposed to blue light (BL), where cryptochromes would be expected to dominate the response, all six lines developed green, open cotyledons with a shortened hypocotyl. However, the reduction of hypocotyl length among these seedlings was slightly different and the *GmPHYB1* overexpressors produced the shortest hypocotyl (Figure 5A, 5D).

Under red light (RL) (Figure 5A and Figure 5E), the *phyB* mutant developed a long hypocotyl along with green, but non-expanded cotyledons (Figure 5A, 5E). Col-0 seedlings produced a shorter hypocotyl than those of the *phyB* mutant. The overexpression of *GmPHYB1* or *AtPHYB* greatly shortened hypocotyl length and expanded the size of the cotyledons (Figure 5A, 5E). The results proved that *GmPHYB1*, as *AtPHYB*, mediated de-etiolation responses under RL.

Under white light (WL), hypocotyl length was comparable with that produced under RL, implying that *PHYB* might play a major role in controlling hypocotyl length under WL. Furthermore, cotyledon size under WL was larger than when the seedlings were exposed to monochromatic light (FR, BL or RL), suggesting that PHYs and cryptochromes act additively in regulating cotyledon growth.

### *GmPHYB1* regulates root elongation in *A. thaliana* under red light

**Figure 6:**
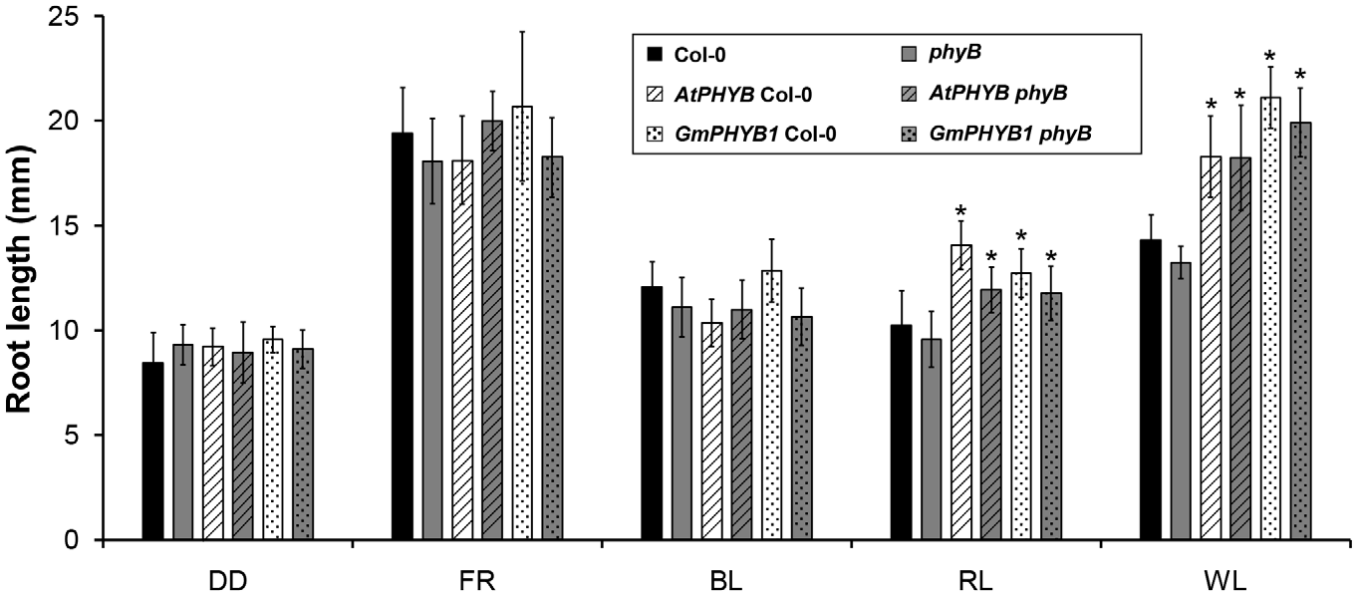
Root length of transgenic plants under different light qualities. Five-day-old seedlings of Col-0, *phyB*, *AtPHYB* Col-0, *AtPHYB phyB*, *GmPHYB1* Col-0 and *GmPHYB1 phyB* grown under DD, FR (23.5 µmol m^−2^ s^−1^), RL (1.3 µmol m^−2^ s^−1^), BL (3.1 µmol m^−2^ s^−1^) or WL (80.8 µmol m^−2^ s^−1^) condition. Asterisks indicated a 0.01 significant difference from its transgenic background (t-test). Error bars denote the standard deviation. n = 15.

PHY exerts a strong influence on primary root elongation in *A. thaliana*, as mutants lacking *PHYB* have reduced primary root elongation [31], [32], [33]. Seedlings of the six lines exposed to either darkness (DD), far-red (FR) or blue light (BL) did not vary with respect to root length. However, exposure to either red (RL) or white light (WL) reduced root length in the *phyB* mutant, while the overexpression of *GmPHYB1* as well as *AtPHYB* markedly increased root length (Figure 6). The observation supported the notion that *GmPHYB1* was also involved in root elongation under RL.

### The effect of *GmPHYB1* overexpression on the response to light fluence rate

Based on the radiation energy of light, phytochrome responses have been subdivided into different classes including low fluence response (LFR), very low fluence response (VLFR), high irradiance response to red light (HIR-R) and high irradiance response to far-red light (HIR-FR), and *PHYB* is mainly responsible for the LFR and HIR-R during photomorphogenesis [34], [35], [36], [37].

**Figure 7:**
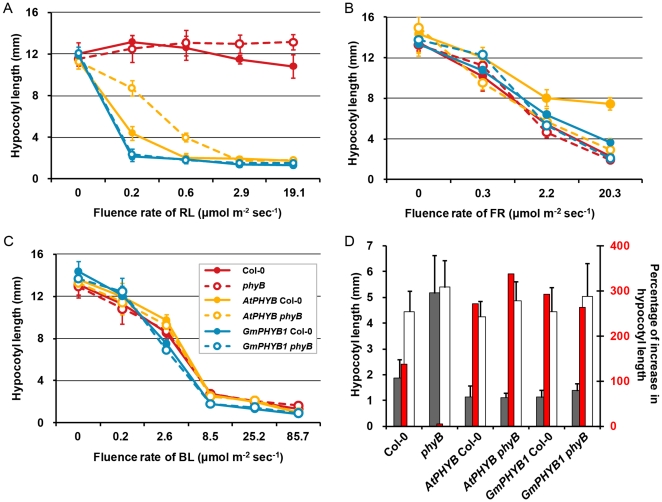
Light fluence response curves of transgenic plants. (A-C) Hypocotyl length of five-day-old seedlings of Col-0, *phyB*, *AtPHYB* Col-0, *AtPHYB phyB*, *GmPHYB1* Col-0 and *GmPHYB1 phyB* grown under different fluence rate of RL (A), FR (B) or BL(C) conditions. (D) EOD-FR responses of Col-0, *phyB*, *AtPHYB* Col-0, *AtPHYB phyB*, *GmPHYB1* Col-0 and *GmPHYB1 phyB*. Gray columns represented the hypocotyl length of 4-day-old seedlings grown under 8h-day/16h-night cycle; white columns represented hypocotyl length of four-day-old seedlings under the same short day conditions with an additional 15 min FR treatment at the end of the light (left axis). Red columns represented the percentage of increasement in the hypocotyl length of the treated seedlings compared with the untreated seedlings (right axis). Error bars denote the standard deviation. n = 15.

We first examined the *PHYB* HIR-R by measurement of hypocotyl length of 5-day-old seedlings grown under a range of continuous RL fluence rate (Figure 7A). Hypocotyl elongation in the *GmPHYB1* overexpressors was strongly inhibited at a fluence rate of just 0.2 µmol m^−2^ s^−1^ (Figure S3A), while neither *AtPHYB* Col-0 nor *AtPHYB phyB* were inhibited until the fluence rate reached 0.6 and 2.9 µmol m^−2^ s^−1^, respectively. In contrast, hypocotyl elongation in *phyB* seedlings appeared to be independent of light fluence rate.

Exposure to either FR (Figure 7B) or BL (Figure 7C) inhibited hypocotyl elongation of the overexpressors of *GmPHYB1* or *AtPHYB* as the light fluence rate was increased. At 20.3 µmol m^−2^ s^−1^ of FR, *AtPHYB* Col-0 seedlings developed a longer hypocotyl than those of the other five lines (Figure S3B), which had been observed in Figure 5A and Figure 5C. Additionally, hypocotyl elongation of the two *GmPHYB1* overexpressors was clearly inhibited (2/3 length of Col-0 or *phyB*) when the fluence rate of BL reached 8.5 µmol m^−2^ s^−1^ (Figure S3C), though hypocotyl elongation of the *AtPHYB* overexpressors did not appear to be so strongly inhibited by BL.

*PHYB*-mediated LFR was assessed by measuring hypocotyl length following an end-of-day FR (EOD-FR) treatment [38]. The hypocotyl of Col-0 seedlings showed a marked response to this treatment (Figure 7D), and the effect was even more pronounced in the *GmPHYB1* and *AtPHYB* overexpressors, presumably due to the abundance of PHYB in these seedlings. In contrast, *phyB* mutant had no such response to the treatment.

### The altered expression of genes related to flowering time & hypocotyle elongation in *GmPHYB1* overexpressor

To study the mechanism of the overexpression of *GmPHYB1* on early flowering under SD and the strong inhibition of hypocotyl elongation, the expression of a set of key flowering time genes and hypocotyl growth related genes was monitored by qPCR.

**Figure 8:**
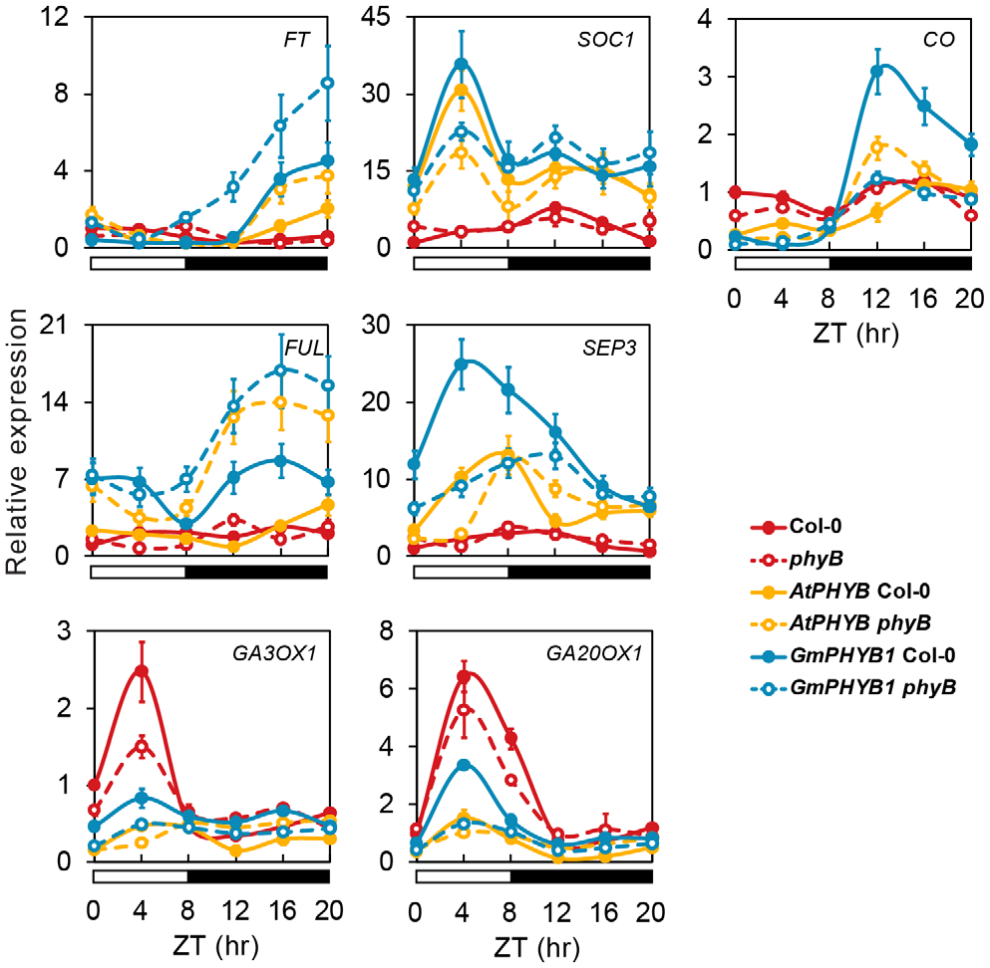
The effect of overexpression of *GmPHYB1* on the expression of genes related to flowering time and hypocotyl elongation. The diurnal variation in transcript profiles of *FT*, *SOC1*, *CO*, *FUL*, *SEP3*, *GA3OX1* and *GA20OX1* in 10-day-old seedlings of Col-0, *phyB*, *AtPHYB* Col-0, *AtPHYB phyB*, *GmPHYB1* Col-0 and *GmPHYB1 phyB* grown under SD conditions, as detected by qPCR. Error bars denote the standard deviation.

Under SD conditions, transcript abundance of *FT* (*FLOWERING LOCUS T*), *SOC1* (*SUPPRESSOR OF OVEREXPRESSION OF CONSTANS 1*) and *CO* (*CONSTANS*) [39] in all of the transgenic lines was elevated, while *FUL* (*FRUITFULL*) and *SEP3* (*SEPALLATA3*) [40], [41] were also regulated in the overexpressors except in *AtPHYB* Col-0 (Figure 8). These results were basically in agreement with the phenotype observed above. The flowering time of Col-0 and *phyB* was delayed under SD, *AtPHYB* Col-0 had an intermediate flowering time, while the flowering of the other transgenics was accelerated.

There were no regular changes in any of the hypocotyl growth related genes tested (*PIF*s and *COP*s, data not shown). However, a decline was noted in the rhythmic amplitude of the expression of *GA3OX1* and *GA20OX1* (both of which are involved in gibberellin synthesis) in the presence of overexpression of *GmPHYB1* as well as *AtPHYB* (Figure 8). Some evidence has been presented to suggest that in light-grown plants, PHYs can inhibit the synthesis or action of gibberellin, which was known to stimulate hypocotyl elongation[42]. Any interference in gibberellin synthesis which reduces the quantity of endogenous gibberellin may thus at least in part explain the observed inhibition of hypocotyl elongation in the transgenic plants.

### *AtPHYA* is down-regulated in *AtPHYB* Col-0

**Figure 9:**
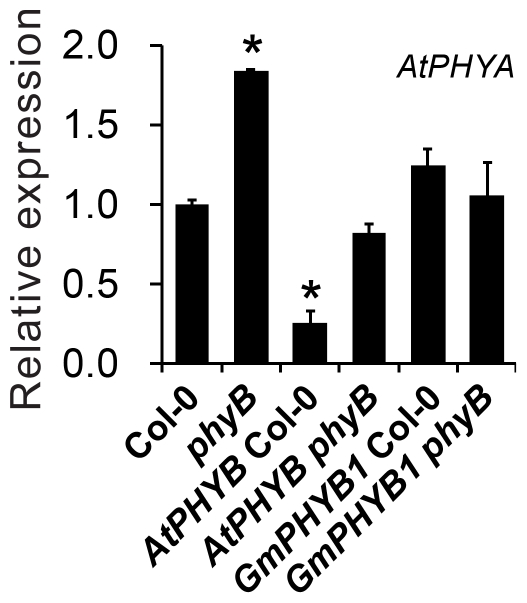
The expression abundance of *AtPHYA* in transgenic *A. thaliana.* Transcription of *AtPHYA* in 10-day-old seedlings of Col-0, *phyB*, *AtPHYB* Col-0, *AtPHYB phyB*, *GmPHYB1* Col-0 and *GmPHYB1 phyB* grown under SD conditions, as detected by qPCR. Asterisk indicates a 0.01 significant difference from Col-0. Error bars denote the standard deviation.

*AtPHYB* Col-0 seedlings exhibited longer hypocotyls under far-red light (Figure 5A, 5C, 7B). To test whether this behavior reflected the altered expression of *AtPHYA*, its transcript abundance was monitored using qPCR. As Figure 9 showed, *AtPHYA* was up-regulated in the *phyB* mutant, and down-regulated in *AtPHYB* Col-0. This phenomenon of elongated hypocotyls under FR resulting from overexpression of *PHYB* had been previously demonstrated [43], [44], [45]. However, the expression of *AtPHYA* was not affected by the overexpression of *GmPHYB1*, suggesting a functional difference between *GmPHYB1* and *AtPHYB*.

## Discussion

In our study, a *PHYB* homolog, designated as *GmPHYB1*, was isolated in soybean, corresponding to the genomic locus Glyma09g03990. A second locus showing appreciable sequence similarity to *PHYB* was Glyma15g14980. This locus is quite likely a homolog of *GmPHYB1*, since soybean is a paleopolyploid [16], [46], in which a good many genes are represented by more than one copy [47]. Supporting the hypothesis, the two soybean *PHYB*-like genes were located in two distinctive chromosomes (No. 9 & 15). So far, multiple copies of *PHY* homologs are known in the genome of maize, potato, tomato and poplar [19], [22], [48], [49]. Four *PHYA* homologs were found in soybean genome [14]. Two *PHYA* genes *GmPHYA2* [14] and *GmPHYA3* [15] have been functionally identified in the soybean genome, which correspond to two previously identified maturity loci, *E4* and *E3*, respectively. *GmPHYA1* gene was also isolated, while its function remained undetermined. However, both the protein sequences and the gene expression patterns suggested that *GmPHYA1* might have a function similar to that of *GmPHYA2* [14].

The *GmPHYB1* sequence shows a high level of conservation with other plant *PHYB* sequences. Its exon/intron structure is identical to that of its *A. thaliana* and rice homologs [21], though the soybean introns were generally longer than those in *A. thaliana* (Figure 1B). The length of its gene product (1,137 residues) was consistent with the length of PHYB proteins characterized to date, and its level of peptide similarity with AtPHYB was over 70% (over 80% with PHYBs from other legume species). Finally, the GmPHYB1 protein included all the known conserved functional PHY domains, namely P1 (Gly/Ser-rich), P2/PAS, P3/GAF, P4/PHY, PAS1, PAS2, histidine kinase and a chromophore-binding site [4] (Figure S1).

In soybean, *GmPHYB1* was expressed in the full range of tissues/organs sampled and at every developmental stage studied (Figure 2). Its overall level of expression in the aerial part of the plant increased as development proceeded (Figure 2B), but varied from organ to organ; in the unifoliolates, for example, expression peaked at the time of flowering, while in the trifoliolates, the peak occurred before flowering (Figure 2B). The implication is that *GmPHYB1* is likely involved in a number of developmental processes, with each organ associated with a characteristic pattern of expression, reflecting the protein's specific function.

Photoreceptors are critical molecules that function at the interface between the organism and environmental cues, providing plants with signals for the control of germination, seedling development, shade avoidance, plant architecture, and photoperiodic flowering [7], [50], [51]. The ectopic expression of *GmPHYB1* in *A. thaliana* had a similar, but not fully identical effect on aspects of growth and development as produced by the overexpression of the native copy of *PHYB*. Thus, for example, the *GmPHYB1* overexpressors displayed typical *PHYB*-related and red light specific phenotypes. The overexpression of *GmPHYB1* (and the overexpression of *AtPHYB*) induced a similar expression pattern of genes related to flowering time and hypocotyl growth, suggesting that these two genes operate in a very similar manner to one another. Nevertheless, there were a number of subtle distinctions between the *AtPHYB* overexpressors and the *GmPHYB1* overexpressors. Firstly, the latter induced a higher transcript abundance of the flowering time associated genes of *CO*, *FT*, *SOC1*, *FUL* and *SEP3* (Figure 8), which might result in a more pronounced acceleration in flowering time under SD conditions (Figure 4). Secondly, a much lower fluence rate of red light was sufficient to induce hypocotyl shortening in the *GmPHYB1* overexpressors (Figure 7A). Thirdly, the length of the hypocotyl formed by the *GmPHYB1* overexpressors was much less than that formed by the *AtPHYB* overexpressors either when the fluence rate of blue light used was 8.5 µmol m^−2^ s^−1^ or 25.2 µmol m^−2^ s^−1^ (Figure 7C, S3C). The latter observation was suggestive of an interaction between PHY and the cryptochromes in the control of hypocotyl growth [52], [53], [54]. It is well known that soybean is a heliophilous plant, while *A. thaliana* is a skiophilous one [55]. Thus it is likely that the GmPHYB1 protein is more adapted to conditions of stronger incident radiation than AtPHYB; this might explain why the overexpression of *GmPHYB1* in *A. thaliana* had a greater phenotypic effect than that of *AtPHYB* did. Finally, it is also possible that the interaction between PHYB and PHYA may differ qualitatively between soybean and *A. thaliana*, because the overexpression of *AtPHYB* down-regulated *PHYA* expression in Col-0, while the ectopic expression of *GmPHYB1* did not (Figure 9).

It has been proposed that the function of PHYB is universal among flowering plants [11]. The present data show that GmPHYB1 has much in common with AtPHYB, though there are subtle differences between them related to phenotypic intensity (e.g., earlier flowering under SD conditions and shorter hypocotyl length under certain light fluence rate) when the two genes were expressed in *A. thaliana*. A more precise determination of the function of GmPHYB1 will require experiments based on a transgene where the gene is driven by its native promoter in soybean. It would be of interest to determine the function of the second soybean *PHYB* gene, and possible interaction between the two.

## Materials and Methods

### Plant materials and growing conditions

The endogenous expression profiles of *GmPHYB1* within various tissues/organs and at various developmental stages of the soybean cultivar Kennong18 were obtained from RNA harvested from plants raised at 28°C, with an 8h-light/16h-dark photoperiod, where the light fluence rate was 100–150 µmol m^−2^ s^−1^. Seedlings were harvested before the expansion of the unifoliolates leaves. Various tissues/organs including unifoliolates, trifoliolates, hypocotyls, epicotyls, cotyledons, petioles, shoot apex (including the apical meristem and immature leaves), stems, flowering buds, and the whole aerial organs of plants were individually sampled when the unifoliolates, the 1^st^, 2^nd^, 3^rd^ or 4^th^ trifoliolates had fully expanded or when plants reached flowering. Pods without seeds were sampled at 7, 14, and 21 days after flowering and at maturity.

### Gene isolation, plasmid construction and production of transgenic *A. thaliana* plants

The fragment containing the coding sequence of *GmPHYB1* was amplified with a pair of gene-specific primers (Table S1), and was then introduced into the pEASY-T1 vector (TransGen Biotech, CHN). Several independent clones were sequenced for *bona fide* sequence. The coding sequence of *GmPHYB1* was subsequently cloned into entry vector pDONR207 (Invitrogen) and finally transferred to the expression vector pLeela via the LR Gateway recombination reaction (Invitrogen). *A. thaliana* plants were agroinfected with *Agrobacterium tumefaciens* strain pGV3101 MP90RK, using the floral dipping method [56]. Basta-resistant T_1_ plants were self-pollinated through four generations to obtain the homozygous T_4_ transgenic lines for analysis.

### Measurement of flowering time, hypocotyl and root length and EOD-FR experiment

At least three independent lines per transgene/background combination were assayed (although the data for just one has been presented here for clarity). Measurement of flowering time and hypocotyl and root length of *A. thaliana* was performed as describe by Xiao *et al*. [57]. EOD-FR experiment followed the procedure elaborated by Robson *et al*. [58].

### Quantitative real-time RT-PCR

Quantitative real-time RT-PCR (qPCR) with gene-specific primers (Table S1) was implemented using an ABI StepOne Real-Time PCR system (Applied Biosystems, USA), based on SYBR Premix ExTaq polymerase (TaKaRa, Japan). Expression data were analyzed using StepOne software (ABI, Applied Biosystems, USA) and transcript levels were calculated relative to the corresponding reference gene (Table S1). For the tissues/organs and developmental samples of soybean, the reference gene was *GmUKN1* [59] and for the gene expression in transgenic *A. thaliana*, it was *At4g34270* [60]. Two lines per each background in which *GmPHYB1* was overexpressed were monitored (although for clarity only one line per background is presented here).

## Supporting Information

Figure S1 **Multiple alignment of the complete sequences of AtPHYB (P14713) and GmPHYB1 (EU428749).** The colored underlines indicate the functional domains of PHY. The asterisk indicated the chromophore-binding site. Shaded residues indicate amino acids matching the consensus.
(TIF)

Figure S2 **Expression levels of** ***GmPHYB1*** **and** ***AtPHYB*** **in the plants studied.** 10-day-old seedlings of Col-0, *phyB*, *AtPHYB* Col-0, *AtPHYB phyB*, *GmPHYB1* Col-0 and *GmPHYB1 phyB* grown under SD conditions, as detected by qPCR. Error bars denote the standard deviation.
(TIF)

Figure S3 **The phenotype of seedlings grown under different light fluence rate.** Five-day-old seedlings (from left to right: Col-0, *phyB*, *AtPHYB* Col-0, *AtPHYB phyB*, *GmPHYB1* Col-0, *GmPHYB1 phyB*) grown under 0.2 µmol m^−2^ s^−1^ RL (A), in 20.3 µmol m^−2^ s^−1^ FR (B), or in 8.5 µmol m^−2^ s^−1^ BL (C). Bar  =  0.5 cm.
(TIF)

Table S1 **Primers used in this work.**
(XLS)
