# Supplementary figures and images for: Ectopic Expression Reveals a Conserved PHYB Homolog in Soybean
Source: PLoS One. 2011 Nov 16;6(11):e27737. doi: 10.1371/journal.pone.0027737 (PMC3218029; doi:10.1371/journal.pone.0027737)

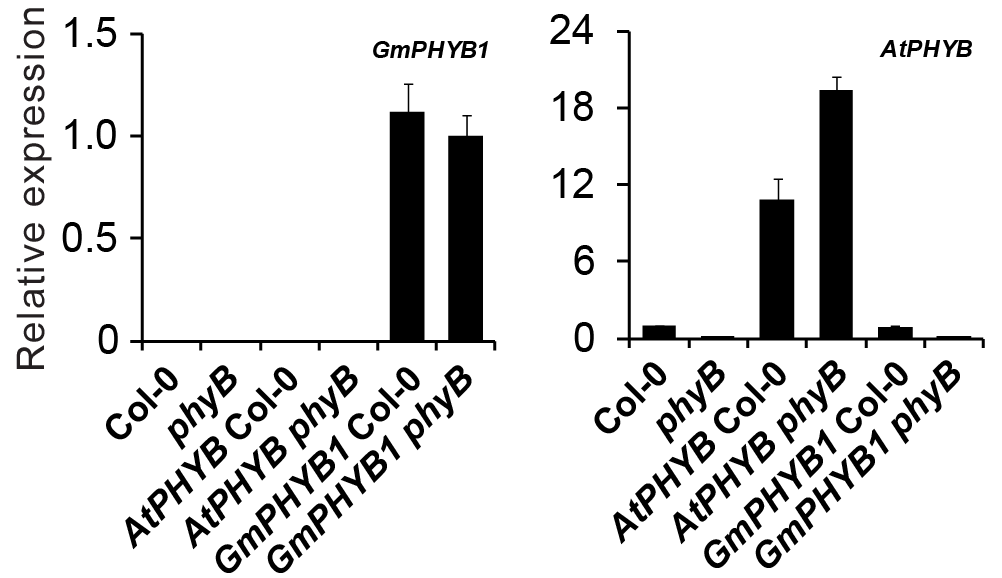

Supplement: Figure S2 — Expression levels of GmPHYB1 and AtPHYB in the plants studied. 10-day-old seedlings of Col-0, phyB, AtPHYB Col-0, AtPHYB phyB, GmPHYB1 Col-0 and GmPHYB1 phyB grown under SD conditions, as detected by qPCR. Error bars denote the standard deviation. (TIF) [file pone.0027737.s002.tif]

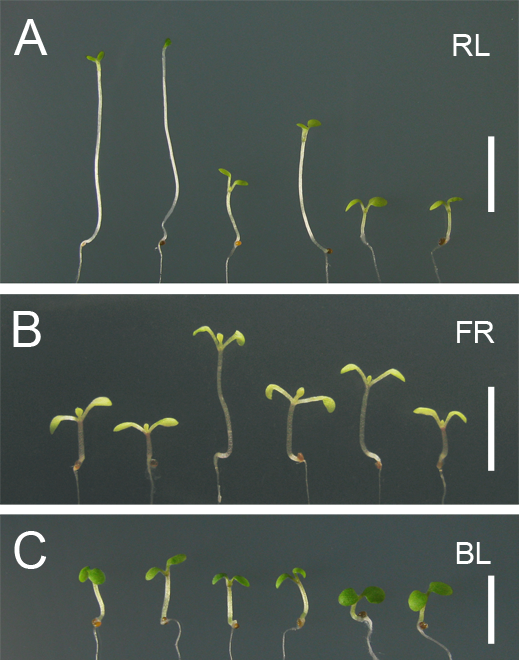

Supplement: Figure S3 — The phenotype of seedlings grown under different light fluence rate. Five-day-old seedlings (from left to right: Col-0, phyB, AtPHYB Col-0, AtPHYB phyB, GmPHYB1 Col-0, GmPHYB1 phyB) grown under 0.2 µmol m−2 s−1 RL (A), in 20.3 µmol m−2 s−1 FR (B), or in 8.5 µmol m−2 s−1 BL (C). Bar = 0.5 cm. (TIF) [file pone.0027737.s003.tif]
